# Introduction of a Zn-based metal–organic framework @ biomass porous activated carbon as a high-sensitive coating for a stainless steel SPME fiber: application to the simultaneous analysis of nonsteroidal anti-inflammatory drugs

**DOI:** 10.1186/s13065-022-00818-w

**Published:** 2022-04-05

**Authors:** Yalda Pasandideh, Habib Razmi

**Affiliations:** Department of Chemistry, Faculty of Basic Sciences, Azarbaijan Shahid Madani University, P.O. Box: 53714-161, Tabriz, Iran

**Keywords:** Nonsteroidal anti-inflammatory drugs, Bread hydrochar, High performance liquid chromatography, Hydrothermal carbonization, Solid phase microextraction

## Abstract

The present study introduces a high-efficiency nanocomposite material featuring a zinc-based metal–organic framework and a novel porous activated carbon derived from bread waste. The prepared nanocomposite, namely Zn-MOF-5@BHPAC, has been synthesized by a low-temperature hydrothermal process and coated onto the surface of a stainless steel wire with epoxy glue. The fabricated fiber has been employed as an SPME fiber applied in the extraction and pre-concentration of some nonsteroidal anti-inflammatory drugs (NSAIDs) before their high-performance liquid chromatography-ultraviolet (HPLC-UV) studies. The characterization studies were performed utilizing field emission scanning electron microscopy, elemental mapping, energy-dispersive X-ray spectroscopy, elemental analyzer, Fourier-transform infrared spectroscopy, and Brunauer–Emmett–Teller surface area analysis. Under the optimal conditions, the method demonstrated low detection limits (LODs, 0.06–0.15 µg L^–1^), wide linear ranges (LRs, 0.20–380 µg L^–1^) with good linearity (R^2^ > 0.991), good precisions (RSDs < 6.95%), and acceptable relative recoveries (RR > 85%). Using the green and affordable biomass of bread as a novel carbon-rich source is an innovative idea provided in this study. In addition, the hybridization of the obtained carbon-based material with the MOF compound to create a new high-capacity sorbent is another strength of the proposed method. Long service lifetime, economic efficiency, environmental friendliness, and high extraction capability were some of the other advantages of the suggested procedure. Therefore, the method can utilize successfully for the simultaneous determination of NSAIDs (as model analytes) in different matrixes.

**Supplementary Information:**

The online version contains supplementary material available at 10.1186/s13065-022-00818-w.

## Introduction

Solid-phase extraction (SPME) is an efficacious sample preparation technique that integrates sampling, extraction, enrichment, preconcentration, and isolation of analytes in a single step [1]. In SPME, the analytes are adsorbed from the liquid or gaseous samples onto a solid/polymeric-coated fused silica fiber [2]. The SPME potential and its main advantages (simplicity, low price, compatibility with different analytical systems, automation, and solvent-free extraction) cause special attention to applying it in micro-pollutants analysis [3]. Despite the mentioned benefits, this method possesses some weaknesses, including fragility, bending of the fiber needle, limited numbers of commercially available sorbents, thermal instability, swelling and stripping of coating materials, and low extraction capability [4]. Consequently, different specialized solutions have been suggested to overcome these concerns such as electrochemical and physical coating technologies, employing flexible metallic substrates, and utilizing new adsorbents (for example carbonaceous materials and pristine biomaterials) [5, 6].

Biomass is a renewable organic waste that is increasingly used as a feedstock for liquid biofuels and green bio-based chemicals [7]. All the biologically products such as wood and wood wastes, crops and their residues, municipal solid wastes (MSW), animal manures, industrial wastes, food processing wastes, aquatic plants, and algae are considered biomass [8]. Biochars are a kind of porous carbon-rich materials efficiently produced by biomass wastes [9]. Biochars offer different applications through theirs large surface area, high porosity, functional groups, high cation exchange capacity, and high stability [10]. Direct combustion, pyrolysis, hydrothermal carbonization (HTC), gasification, and torrefaction methods employ for producing a biochar from different biomass sources [11]. Among these methods, pyrolysis and hydrolysis are more efficient. The biochars produced by dry pyrolysis at high temperatures (> 400 °C) and low water content are called pyrochars. In contrast, the biochars produced via the HTC process at relatively low temperatures (180–350 °C) and under autogenous pressure (2–5 MPa) are named hydrochars [12]. Hydrochars possess remarkable advantages over pyrochars, including low energy consumption, faster processing time, high conversion efficiency, and environmentally friendliness [13]. For that reason, the HTC technique has more attention recently. Hydrolysis, dehydration, decarboxylation, and aromatization reactions that occur during the HTC process represent an important role in decreasing the hydrogen/carbon (H/C) and oxygen/carbon (O/C) ratios and generating a carbon-rich product [14]. The HTC products mainly consist of a solid residue (as the main product, hydrochar), gas, and liquid phase productions. Feedstock and the applied method conditions strongly affect the distribution and properties of the final products [15]. Up to now, different feedstocks have been studied with the HTC process, such as cellulose, fructose, glucose, starch, protein, carbohydrates, and food wastes [16]. However, many compounds and wastes have not yet been examined, including bread. Bread is a widely used food that has been the people’s staple meal since immemorial times. Bread is not only one of the most favorite foods, but it is also one of the most-wasted foodstuffs in the world. Unfortunately, about 25% of the total bread and bakery productions are wasted daily and go out of the direct consumption cycle. Bread is mainly composed of carbohydrates (starch), protein (gluten: gliadin, glutenin), cellulose, fat, minerals (calcium, potassium, phosphorus, iron, zinc, and so on), B vitamins, and occasionally phenolic compounds and acrylamide [17]. The presence of such compounds in the bread structure makes it a good choice for the HTC process, which has not been studied before.

Nowadays, carbonaceous materials (that are mostly carbonized from the organic precursors) utilize in adsorption, catalysis, batteries, fuel cells, drug delivery, super-capacitors, and imaging. However, the applications of these materials have been limited because of their low surface areas, displaced structures, and non-uniform sizes [18]. Metal–organic frameworks (MOFs) are a novel class of highly-porous crystalline organic–inorganic hybrid-materials formed by organic linkers and positively charged metal ion groups [19]. The combination of inorganic and organic groups create high-porosities and high specific surface area and accordingly makes the MOFs usable in the adsorption, energy storage, separation and purification, catalysis, and sensing procedures [20].

Recently, researchers discovered that the carbonaceous materials derived/hybrid from/with MOFs could overcome most of these weaknesses [21]. It is demonstrated that the incorporation of carbonaceous materials (such as GO and activated carbon (AC)) into the frameworks of MOFs has improved the stability, porosity, and adsorption attributes of the MOFs [22, 23]. MOFs are good options for the chemical adsorption of smaller molecules, due to the strong and selective interactions created between the MOF network and the target analytes [24]. Contrariwise, the activated carbons due to their greater porosity and larger pore diameters are good in the physico-sorption of larger molecules. As a result, the combination of a MOF and activated carbon into a composite form may create the positive properties of both materials. By adjusting the weight proportion of MOF crystals incorporated into the carbonaceous materials, it could be suggested that the composites can be designed to remove and extract a broader range of contaminants and micro-pollutants [25].

The paper presents a novel and high-performance SPME fiber based on a stainless steel wire coated with a new nanocomposite of Zn-MOF-5@bread hydrochar porous activated carbon (Zn-MOF-5@BHPAC) for the first time. Because of the relatively small pore size and the linkages chemistry of the Zn-MOF-5, it was selected for the synthesis of the Zn-MOF-5@BHPAC nanocomposite. In the MOF-5, [Zn4O]^6+^ tetrahedral occupy the corners of a cubic structure, and 1,4-benzenedicarboxylic (BDC) junctions these zinc oxide clusters [26]. In addition, the functional the prepared hydrochar is possibly anchored the zinc oxide clusters. Besides, non-steroidal anti-inflammatory drugs (NSAIDs) are the most used analgesics and antipyretic medicines in medical and veterinary. The continuous release of these toxic compounds into the environment is harmful to human health. Therefore, the fabricated SPME fiber coupled with the HPLC-UV technique was successfully applied for the simultaneous extraction and determination of some of NSAIDs (just as the model analytes) from different standard/real samples.

## Experimental

### Chemical and materials

Ketoprofen, naproxen, diclofenac, ibuprofen, and mefenamic acid (> 98% purity in each cases) as the model analytes were purchased from Sigma-Aldrich (Steinheim, Germany). A stock solution of 100 mg L^−1^ of each drug was prepared in the HPLC grade ethanol and then stored in the refrigerator at 4 °C. Working standard solutions were prepared by the stepwise dilution of the stock with double distilled water (DDW) before utilizing. HPLC grade acetonitrile (> 99.9%) and methanol (> 99.9%) were supplied from Carlo Erba (Val-de Reuil, France). DDW was obtained from Shahid Ghazi Pharmaceutical Co. (Tabriz, Iran). All other analytical grade chemicals and reagents were obtained from Merck (Darmstadt, Germany). Epoxy glue was purchased from a Betonartoom building materials market in Karaj (Iran). Stainless steel wire (0.3 mm in diameter) was bought from BS Stainless Company (United Kingdom).

### Instruments and apparatus

HPLC analyses were carried out on a JASCO (Japan) high-performance liquid chromatograph equipped with an isocratic PU-1580 pump, a UV-1575 ultraviolet detector (JASCO-1575), and a Rheodyne 7725i six-port switching valve (Rheodyne Cotati CA, USA). The HPLC system was controlled by an HSS-2000 pack with a LC-Net II/ADC interface (JASCO) and a BORWIN software (version 1.50). All the separations were accomplished on an analytical ODS-3 column [250 mm × 4.6 mm ID, 5 μm (MZ-Analysentechnik, Germany)] with an ODS-3 guard column (10 mm × 4 mm ID, 5 μm). A 25-μL microsyringe (zero dead volume, Hamilton, Switzerland) was used for the injection of sample solutions. A Bruker D_8_ Advance X-ray apparatus (Bruker AXS, Karlsruhe, Germany), a Tescan mira3 electron microscope (Brno-Czech Republic), an FTIR system (Bruker, Ettlingen, Germany) spectrometer, and a Brunauer–Emmett–Teller (BET) technique with a Gemini^®^ VII 2390 micrometric instrument and N_2_ adsorption–desorption analysis were applied for the characterization investigations. The elemental analysis was achieved utilizing a PerkinElmer 2400 CHNOS analyzer (United States). Besides, a Happybuy hydrothermal synthesis autoclave reactor with PTFE lined vessel (United States), a Beckman GS-6 centrifuge (USA), an ultrasonic device (Falc instrument Srl LBS2, Italy), an Oakton^®^ PH 550 Benchtop pH Meter Kits (France), a AREX-6 hot plate stirrer (VELP Scientifica Srl, Italy), and a Gimette 50 oven (Gima, Italy) were also employed. High purity nitrogen gas (> 99.99%) was used for solvent evaporation. The chromatographic data were attained at laboratory temperature under isocratic conditions. The wavelength of the UV detector was set at 230 nm.

### Preparation of bread hydrochar

The bread sample was air-dried and ground to the small particles with a mortar. The crushed bread and 40% H_2_SO_4_ (1:8, w/v) were poured into a thermal autoclave reactor and hydrothermally carbonized at 190 °C for 8 h. The obtained solid product was collected and repeatedly washed with DDW to neutralize [27]. Finally, that was dried at 100 °C to obtain the dark brown hydrochar of bread (BH).

### Preparation of bread-hydrochar porous carbon

The achieved BH was mixed with 85% H_3_PO_4_ (1:4, w/v) and heated under an N_2_ atmosphere for 1 h (500 °C) [28]. Ultimately, the product has rinsed to neutralize and dried at 100 °C to obtain the final bread-hydrochar porous carbon product (BHPC).

### Preparation of bread-hydrochar activated porous carbon

The resulting porous carbon was poured in 2 M HCl (1:2) and boiled for 1 h. The product was filtered and washed with DDW to neutral pH and then was oven-dried overnight to obtain the activated porous carbon of bread-hydrochar (BHPAC).

### Preparation of Zn-MOF-5@BHPAC nanocomposite

Briefly, 0.67 mmol Zn(NO_3_)_2_·6H_2_O and 0.22 mmol BDC-acid were dissolved in 30 mL of DMF in an autoclave reactor. Then the prepared BHAPC was added to the precursor solution and ultrasonically dispersed. The obtained mixture was heated at 120 °C for 20 h. Then the autoclave was removed from the furnace and cooled down to ambient temperature. The crystalline precipitates Zn-MOF-5@BHPAC were repeatedly washed with DMF, soaked in chloroform for 12 h, filtered, and finally dried in an oven at 100 °C [29].

### Fabrication of the Zn-MOF-5@BHPAC-coated SPME fiber

2 cm of a stainless steel wire was chemically etched with 37% HCl solution for 20 min. The etched part of the wire was washed with methanol and DDW under ultrasonication (5 min) and dried in air. The etched end was vertically dipped in the epoxy glue for 30 s and then withdrew slowly. The excess glue has been scraped with a glass sheet to give a homogeneous glue layer on the wire surface. The glue-coated wire was inserted into a test tube filled with Zn-MOF-5@BHPAC powder and pulled out slowly after 60 s. The sorbent-coated wire was finally placed into an oven at 120 °C to fix the coating material on the SPME fiber surface [30].

### Microextraction procedure

10 mL of standard/sample solution at the concentration of 10 µg L^−1^ was added into a glass vial. The pH of the sample solution has been adjusted at 3 utilizing 1 M HCl. The Zn-MOF-5@BHPAC-coated SPME fiber was directly immersed into the vial for performing the adsorption and extraction procedures. A magnetic stirrer at 800 rpm has been used to speed up the extraction process. After 15 min, the fiber was removed and immediately inserted into 2 mL methanol for the chemical desorption (5 min). In the following, to prevent dilution effects, improve the technique sensitivity, and adapt the desorption solvent with the chromatographic mobile phase, the solution containing analytes has been evaporated under the nitrogen stream to dry [4]. Finally, the dried samples were dissolved in 100 µL acetonitrile and injected into an HPLC-UV device to analyze. A phosphate buffer (0.02 M, pH = 5): acetonitrile mixture (60:40, v/v) at the flow rate of 1 mL min^−1^ has been employed as the HPLC mobile phase for the isocratic elution of the target analytes.

**Fig. 1:**
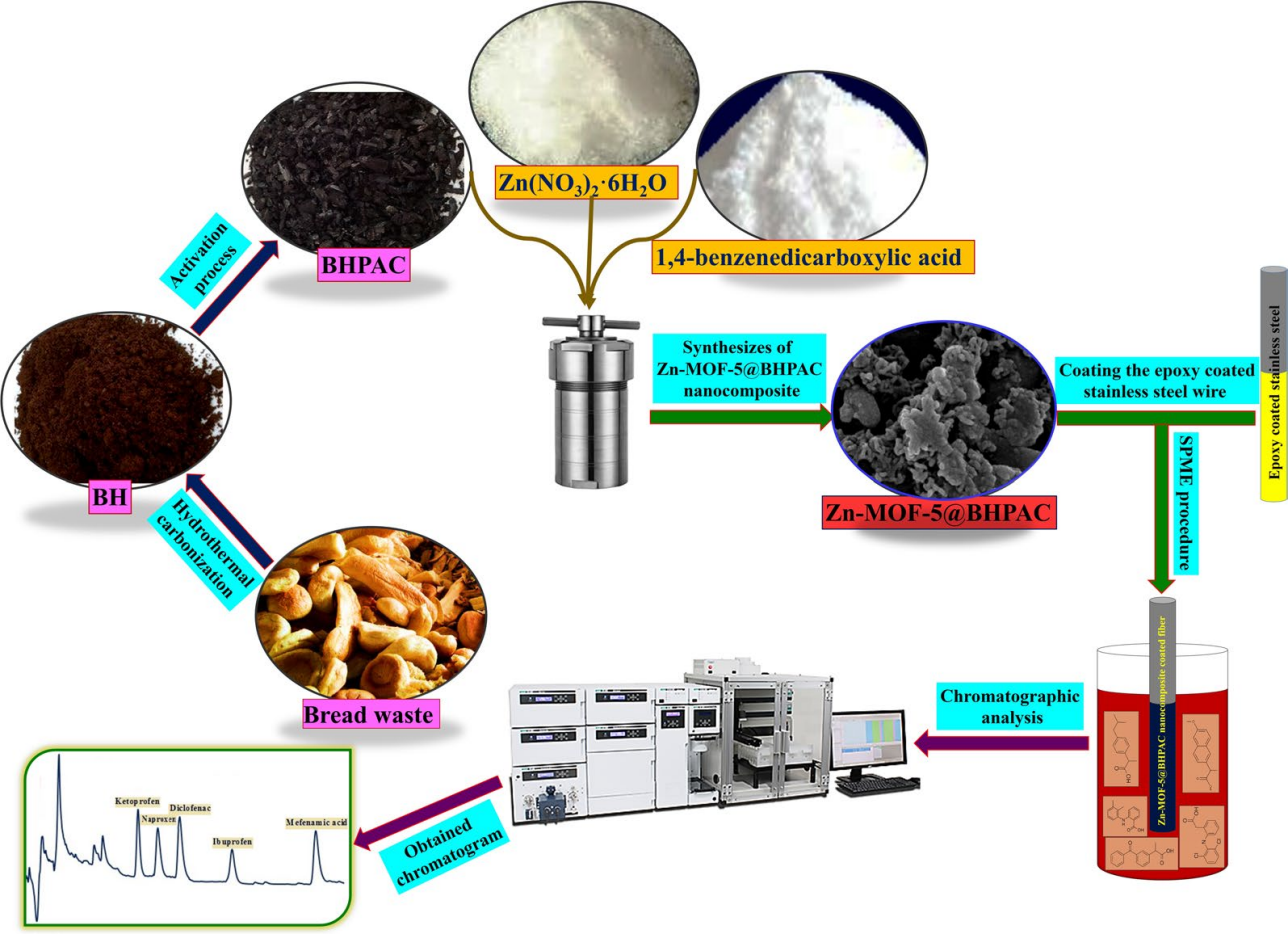
A schematic diagram of the proposed method

A schematic diagram of the proposed method is shown in Fig. 1.

### Real samples preparation

The water samples consisted of powerhouse water (collected from Tabriz thermal power plant, Azarshahr, Iran) and river water (collected from Caspian Sea, Gilan, Iran) samples. The soil samples consisted of high traffic road soil (collected from Shahid Lashkari Highway, near several pharmaceutical factories, Karaj, Iran) and beach soil (collected from Gisum Beach, Gilan, Iran). All the samples were collected in cleaned glass vials and transported to the laboratory in a cooling box. The collected samples were stored in the refrigerator at 4 °C until analysis [31]. The water samples were applied directly for the DI-SPME procedure after filtering through a Whatman Fi filter paper (150 mm), without any other pre-treatment. The soil samples have been firstly air-dried at laboratory temperature. Then 5 g of the dried soil was poured into 10 mL of DDW and ultrasonicated for 3 h. Next, the mixture was centrifuged for 15 min. The overflow solution was utilized for the SPME studies after filtering through a filter paper (150 mm). All the samples were prepared freshly before each analysis.

## Results and discussion

### Characterization of the synthesized materials

**Fig. 2:**
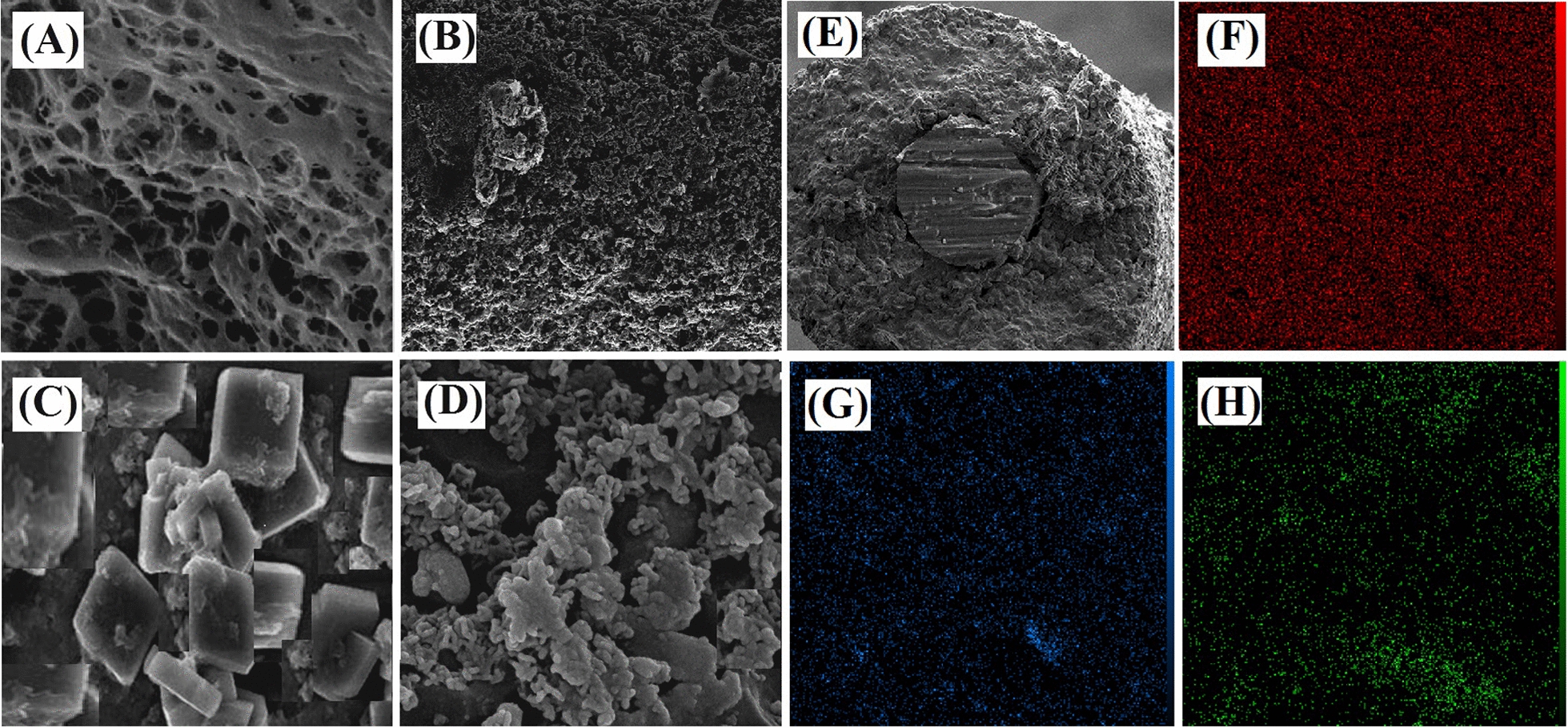
FESEM micrographs of the **A** bread, **B** BHPC, **C** Zn-MOF-5, and **D** Zn-MOF-5@BHPAC nanocomposite, **E** the cross-section micro-image of the proposed SPME fiber and the elemental mapping of the nanocomposite: **F** C, **G** O and **H** Zn

The FESEM, elemental mapping, FTIR, elemental analyzer, and BET techniques have been employed for characterization studies. The FESEM spectroscopy at different magnifications was applied for the surface morphology studies. The standard wheat flour matrix has a fibrous and web-like structure possibly created by gluten protein (Fig. 2A). Irregular and highly porous networks full of pores, cracks, and crevices are observed in the BHPC micro-image (Fig. 2B). In this case, maybe the combination of biopolymers particles during the carbonization procedure has caused the accumulation of several carbon pieces together [32]. In addition, the BH particles have combined during activation, and H_3_PO_4_ was associated with them to form phosphate and polyphosphate bridges [33]. In consequence, the porous structures formed with the addition of phosphate groups during the dilation process. The micro-image of Fig. 2C demonstrates that the synthesized Zn-MOF-5 had a regular cubic structure confirming its successful synthesis. Moreover, the Zn-MOF-5@BHPAC nanocomposite image can be observed in Fig. 2D. It shows that in the Zn-MOF-5@BHPAC hybrid, Zn-MOF-5 is located inside the pores of the BHPAC [25]. The thickness of the Zn-MOF-5@BHPAC nanocomposite layer was 62 µm. A picture of the cross-section of the proposed fiber can be seen in Fig. 2E. In addition, the elemental mapping of the nanocomposite is presented in Fig. 2F, G, H.

**Fig. 3:**
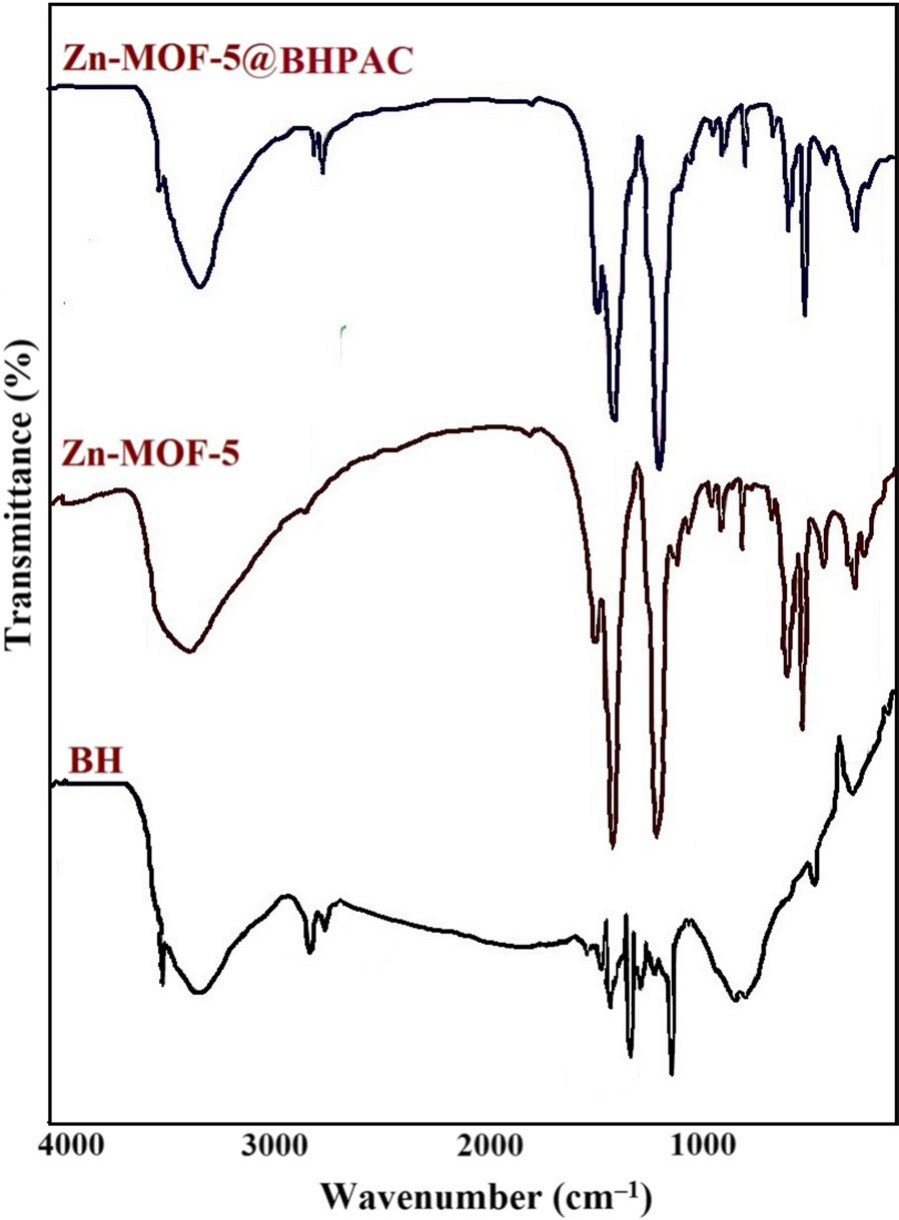
FTIR spectra of the synthesized BH, Zn-MOF-5, and Zn-MOF-5@BHPAC nanocomposite

**Fig. 4:**
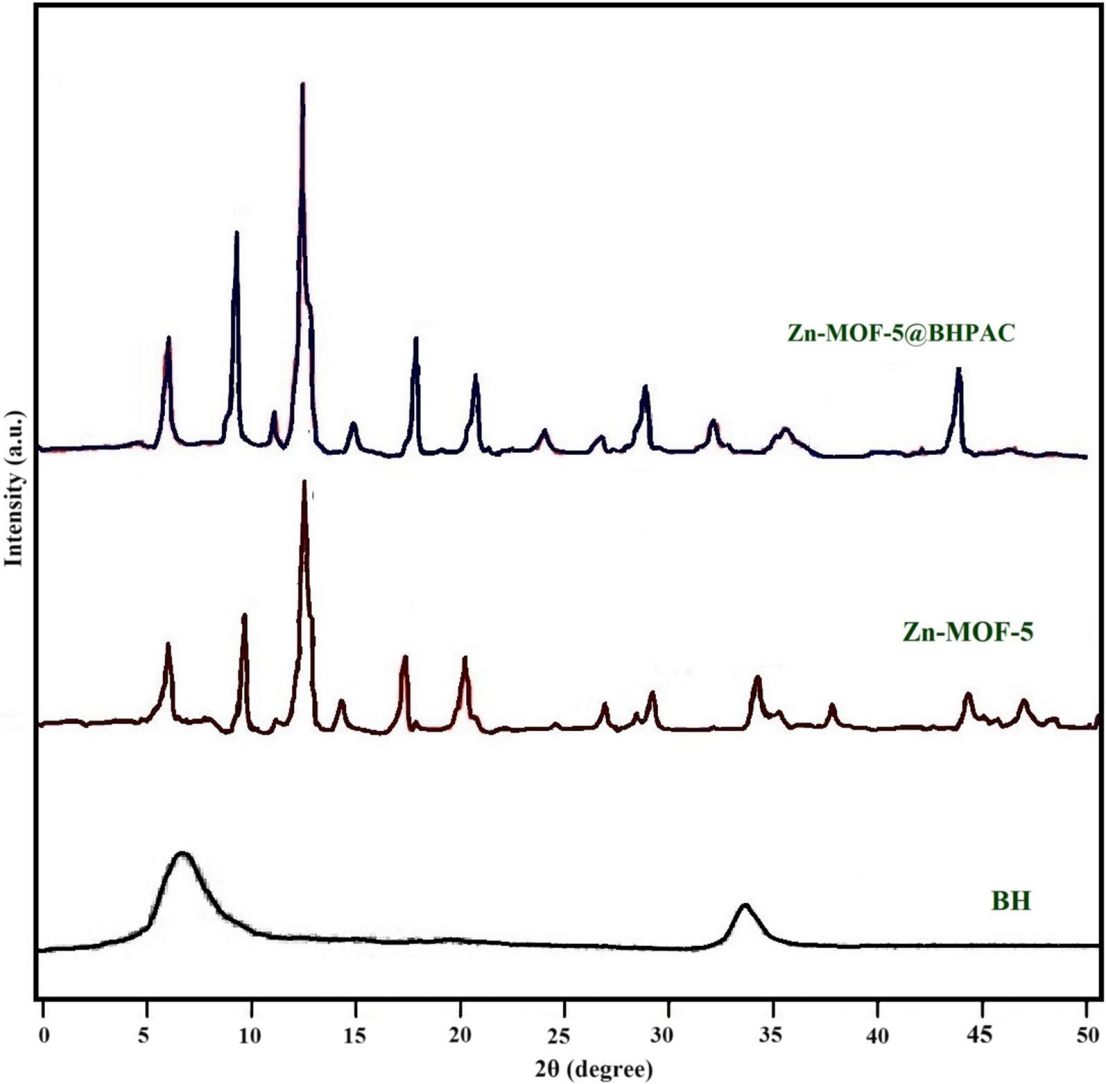
XRD patterns of the synthesized BH, Zn-MOF-5, and Zn-MOF-5@BHPAC nanocomposite

FTIR spectroscopy is a useful tool providing valuable information about the chemical composition and functional groups of compounds. The FTIR spectra of the synthesized BHPAC, Zn-MOF-5, and Zn-MOF-5@BHPAC nanocomposite were recorded over the range of 4000–400 cm^−1^ (Fig. 3) [34]. The FTIR spectrum of the BHAPC has been shown in Fig. 4A. The broadband observed at 3435 cm^−1^ is attributed to the O‒H or N‒H stretching vibrations. The bands at 2920 cm^−1^ and 2846 cm^−1^ are ascribed to the aliphatic C‒H stretching vibrations. The peak that appears at 1649 cm^−1^ is related to the C=O stretching vibrations. The absorption bands at 1450‒1000 cm^−1^ are assigned with the C=O (hydroxyl, ester, or ether), C=C, and O=H stretching vibrations. These results prove that the obtained porous carbon was amorphous without special surface functional groups. The FTIR examination of the synthesized Zn-MOF-5 is presented in Fig. 4B. Two absorption bands at 2950 and 2872 cm^−1^ are attributed to the C‒H stretching vibrations of the methylene groups. The bands at 1700–1400 cm^−1^ are assigned to the carboxylic functionality of the BDC. The peak that appeared at 1760 cm^−1^ is probably related to the C=O stretching vibration of the carboxylate groups. The bands appearing at 1660, 1581, 1540, and 1490 cm^−1^ are attributed to the asymmetric stretching vibration of the C=O group, while its symmetric stretching vibration has emerged at 1396 cm^−1^. The band at 609 cm^−1^ is also ascribed to the symmetric stretching vibration of Zn_4_O. In addition, the broadband at 3000–3605 cm^−1^ is probably related to the presence of water with the metal coordination. The FTIR spectrum of the Zn-MOF-5@BHPAC nanocomposite (Fig. 4C) is displayed that the addition of the presence of BHPAC were not significant changes in the crystalline structure of the nanocomposite. It also proves that the addition of a small number of carbonaceous materials does not affect the properties of MOF compounds [35].

The XRD patterns of the synthesized BHPAC, Zn-MOF-5, and Zn-MOF-5@BHPAC nanocomposite are shown in Fig. 4. The XRD pattern of the BHPAC exhibits two typical diffraction peaks at around 22° and 44° are corresponding to the (002) and (100) crystallographic planes of the graphitic carbon, respectively [36]. Both of these broad peaks indicate an increasing regularity of crystalline structure and better alignment of layers. Since the BHPAC was the host substance, its structural characteristics preserved the dominant position of the nanocomposite substance. After the addition of the Zn-MOF-5, the nanocomposite peaks became split and more obvious. In the other words, no notable changes have occurred by the addition of small values of the carbonaceous material. This result is consistent with the obtained information from FESEM and FTIR analysis.

The elemental analysis of bread, hydrochar, and porous carbon obtained with CHNOS analyzer are listed in Table 1. The bread sample was contained 35.73 wt.% C, 8.56 wt.% H, 0.47 wt.% N, 55.24 wt.% O and 00.00 wt.% S. The BH was contained 64.22 wt.% C, 3.50 wt.% H, 0.27 wt.% N, 32.01 wt.% O and 00.00 wt.% S. The BHPC was contained 78.92 wt.% C, 1.88 wt.% H, 0.37 wt.% N, 18.82 wt.% O and 00.00 wt.% S. As a result, the obtained BHPC is rich in carbon.

**Table 1:** The elemental analysis of the bread, BH, and BHPC

Samples	C (%)	H (%)	N (%)	O (%)	S (%)
Bread	35.73	8.56	0.47	55.24	00.00
BH	64.22	3.50	0.27	32.01	00.00
BHPC	78.92	1.88	0.37	18.82	00.00

**Fig. 5:**
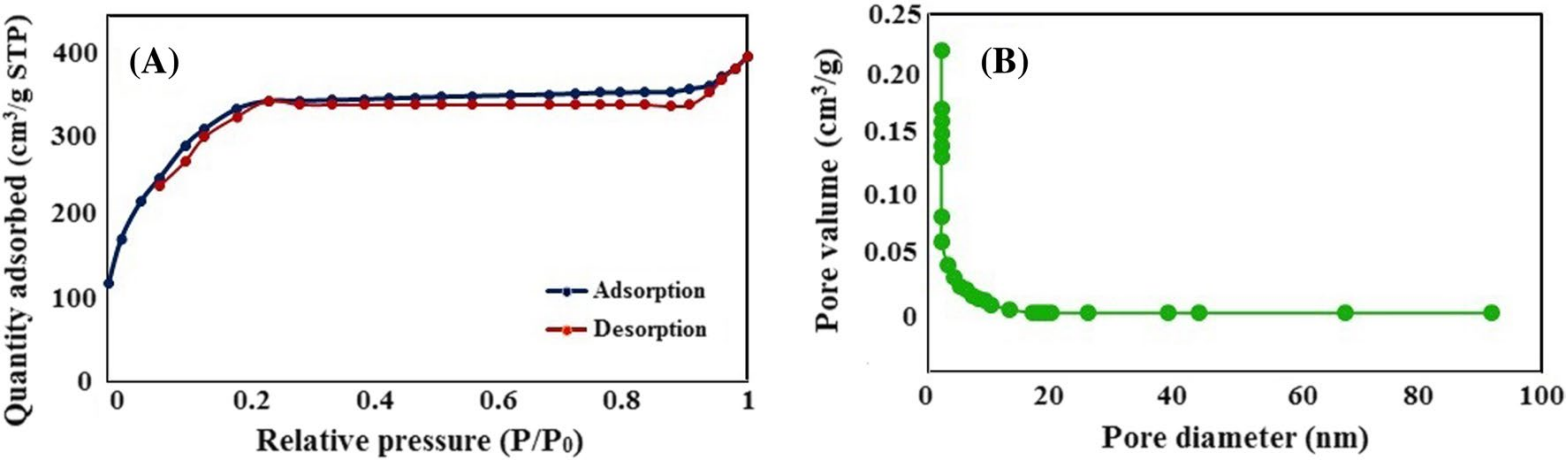
**A** N_2_ adsorption–desorption isotherms) and **B** NLDFT pore-size distribution of the Zn-MOF-5@BHPAC nanocomposite

The N_2_ adsorption–desorption isotherm has been utilized to examine the nanocomposite volume and pore size of the synthesized Zn-MOF-5@BHPAC (Fig. 5). The pore size distribution was calculated by the NLDFT method. The BET analysis data of BHPAC, Zn-MOF-5, and Zn-MOF-5@BHPAC nanocomposite are also summarized in Table 2. A significant porosity reduction in the composite pore size would prove that Zn-MOF-5 crystals were entered inside the BHPAC porosities and caused a partial pore-blocking. In addition, the surface areas of the composites do fall in-between those of the MOF and BHPAC individually [25]. In other words, the higher the MOF content creates the lower the surface area.

**Table 2:** The BET analysis of the prepared BHAC, Zn-MOF-5, and Zn-MOF-5@BHPAC nanocomposite

Sample	BET surface area (m^2^/g)	Average surface area (m^2^/g)	Average pore volume (cm^3^/g)	Average pore size (nm)
BHPAC	2158.109	1650.661	1.237	230.296
Zn-MOF-5	472.388	318.090	0.241	216.830
Zn-MOF-5@BHPAC	306.617	125.508	0.177	151.594

Therefore, according to the results, the high absorption capacity of the synthesized nanocomposite can be mainly attributed to the following factors [37]. (1) Porous structure of hydrochar that is contained macro-pores and meso-pores. These porosities were probably created due to the removal of organic matter, formation of many internal porous structures through the reactions between BH and H_3_PO_4_, dehydration reactions between acid molecules during heating, and release of gaseous products. (2) Π‒Π conjugation effect has been occurred between the benzene ring of ligand and analytes. (3) Coordinating or hydrogen bonds, and poor columbic interaction of analytes and MOFs matrix. (4) Coordination effect has been occurred between the Zn-MOF-5@BHPAC coating and analytes. (5) Simultaneous use of adsorption capability of BHPAC for larger analytes and MOFs for smaller molecules.

Equation 1 determined the yield of the synthesized hydrochar (44.76%).

1 $$Hydrochar\, yield=\frac{Amount\, of\, obtained\, solid\, after\, HTC\, (g)}{Initial\, amount\, of\, bread \,(g)} \times 100$$

### Optimization of the important parameters

The current study has been included two separate steps: (a) manufacture of a high-capacity SPME fiber with a new green bio-based coating material and (b) employing the fabricated fiber in the extraction and determination of some NSAIDs. Therefore, the optimization of important factors influencing both of these platforms was necessary to reach a high-performance microextraction procedure.

**Fig. 6:**
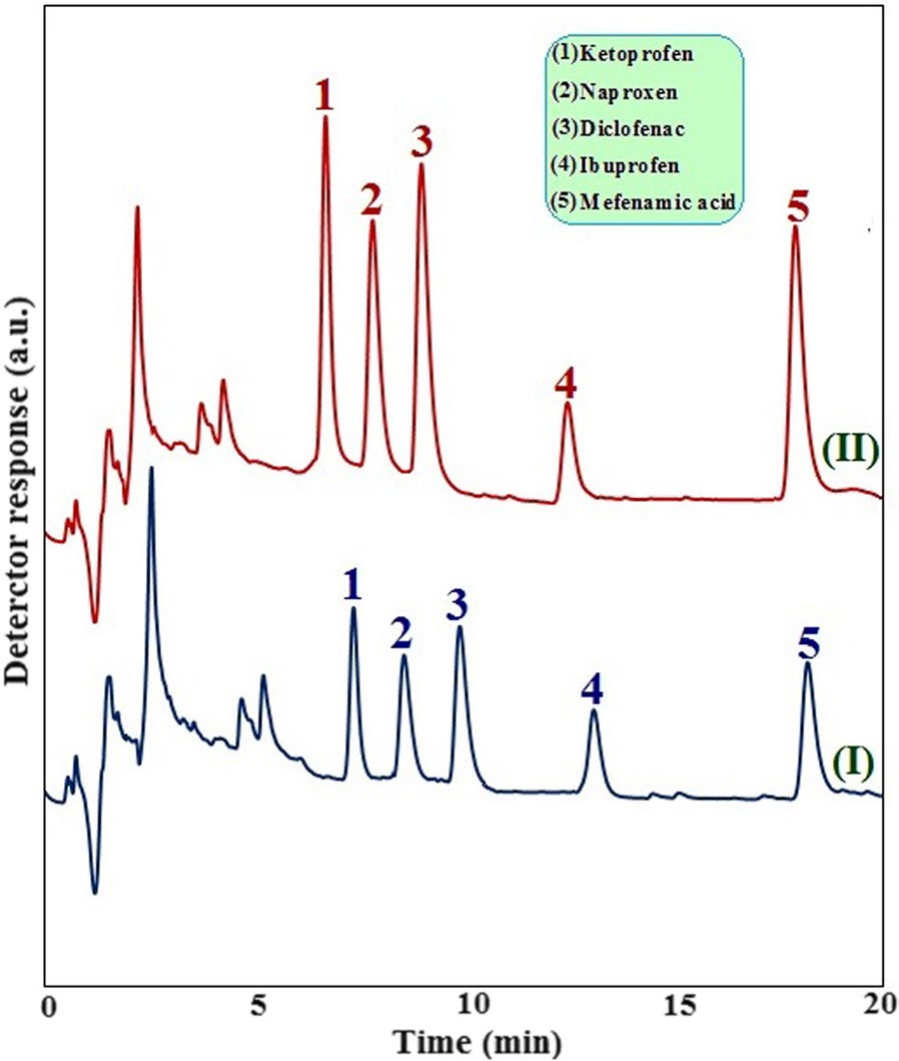
Chromatograms obtained using Zn-MOF-5@BHPAC nanocomposite-coated SPME fiber for road soli sample (I) and the same sample spiked with 25.0 µg L^−1^ of selected NSAIDs (II)

Firstly, the requirements for the fabrication of a good quality SPME fiber have been optimized. In this step, for better comparison and achieving more accurate results, the other analysis parameters have been constant at: the sample volume: 10 mL; sample concentration: 10 µg L^−1^; extraction time: 30 min; desorption solvent: ethanol; desorption solvent volume: 2 mL, desorption time: 15 min and stirring rate: 400 rpm. After fabricating a proper Zn-MOF-5@BHPAC nanocomposite-coated fiber, the significant experimental factors have been optimized. The best factors of each step were employed in the further stages. Figures 4, 5 and 6 express the gradual enhancement of the HPLC signals intensity and the improvement of the technique performance by the optimization of the effective parameters.

#### Optimization of the affecting parameters on the coating quality

Different parameters, including the acid concentration, impregnation ratio (matter: acid), temperature, and time can affect the quality and the major properties of BH, BHPC, and BHAPC. Therefore, all of these parameters were examined in different ranges, and the optimum conditions were obtained as follows:

A. Best conditions for the preparation of BH (Additional file 1: Fig. S1A): H_2_SO_4_ concentration = 40%, bread: H_2_SO_4_ ratio = 1:8 (w/v), T = 190 °C, time = 8 h.
B. Best conditions for the preparation of BHPC (Additional file 1: Fig. S1B): H_3_PO_4_ concentration = 85%, BH: H_3_PO_4_ ratio = 1:3 (w/v), T = 500 °C, time = 1 h.
C. Best conditions for the preparation of BHPAC (Additional file 1: Fig. S1C): HCl concentration = 2%, BHPAC: HCl ratio = 1:2 (w/v), T = laboratory temperature, time = 1 h.
D. Best conditions for the preparation of Zn-MOF-5@BHPAC (Additional file 1: Fig. S1D): T = laboratory temperature, time: 20 min.

#### Kind of fiber coating

To evaluate the extraction capability of the different sorbents, the attained results utilizing the coated stainless steel coated with the BH, BHPC, BHPAC, Zn-MOF-5 and Zn-MOF-5@BHPAC sorbents have been compared with the bare-etched stainless steel wire and the epoxy glue-coated wire (Additional file 2: Fig. S2A). The bare-etched stainless steel wire and the glue-coated one did not present any adsorption and extraction features for the target analytes. However, the improvement of the fibers’ adsorption properties has been obviously observed with changing and modification of the coatings type. As can be found from the Figure, both BHPAC and Zn-MOF-5 alone display acceptable adsorption property, although the adsorption capability of BHPAC is most greater than Zn-MOF-5. This can be attributed to the higher specific surface area and more porosities of BHPAC. But the combination of BHPAC and Zn-MOF-5 creates a composite that has the positive properties of both groups at the same time and creates good practical application via better mechanical-stability and physico/chemically sorption capability [38]. In other hands, the better adsorption capacity of the Zn-MOF-5@BHPAC coated fiber is probably due to the enhanced dispersive forces caused by the new small pores formed at the interface of BHPAC and MOF-5 units [39]. Therefore, highest extraction and adsorption capability was obtained using the Zn-MOF-5@BHPAC nanocomposite sorbent.

#### Chemical etching reagent

Chemical etching is a machining procedure that uses corrosive chemicals (etchants) to create intricate designs on the surface of a metal. The etching is normally employed to dissolve unwanted substances and compose the final design, with no changes in the original material attributes [40]. For the first time in analytical chemistry, Xu and his collaborators used the etching process to make a new SPME fiber. This fiber has been utilized for the isolation and preconcentration of some PAHs from aqueous samples [41]. In general, the etching of a metallic SPME fiber enhances the adhesion between the coating materials and the fiber surface via enhancing the roughness, porosity, and specific surface area and consequently improves the method’s efficiency [42]. In this study, different acid including H_2_SO_4_, HCl, HNO_3_, and HF have been tested to gain the appropriate chemical etching reagent (Additional file 2: Fig. S2B). The best results were obtained utilizing HCl as an ideal etchant. Etching with HCl delivered a porous structure with Cr_2_O_3_, CrF_2_, FeF_3_, and Fe_2_O_3_ and groups on the steel surface, providing high affinity to the coating materials [41].

#### Etching time

Time is a big challenge in the etching process and must be optimized. If the time is not sufficient for the complete material removal, an incomplete-etching will take place and the acceptable roughness and porosity are not probably created. On the opposite, if the time is larger than required, over-etching will happen, and the substrate may be solved or damaged [43]. Here, the etching time impact was examined in the range of 5–45 min. As can be seen in Additional file 2: Fig. S2C, the peak areas developed as the time increased from 5 to 30 min and then gradually decreased. This indicates that over long time, the steel surface degraded and the fiber efficiency reduced as a result of the over-etching process. Therefore, 30 min was set as the suitable chemical etching time.

#### Number of coating layer

The coating thickness determines the equilibrium time and the fiber adsorption capacity. Generally, a thicker geometry film provides higher extraction efficiency and greater sensitivity, but it needs a longer time to reach equilibrium compared to a thinner coating [44]. Therefore, adjusting the thickness of the fiber coating is of particular importance. For investigating the effect of the coating thickness, the glued wires were coated between 1 to 4 times with the prepared nanocomposite. According to the results, the extraction capacity decrease by increasing the coating thickness (Additional file 2: Fig. S2D). It seems that at the first coating step, a uniform coating with a suitable thickness has been created at the fiber surface. The surface porosity or active adsorption sites are probably reduced with more coating layers [45]. Therefore, a 60 s-single-stage coating was selected as the optimal value.

### Optimization of the important experimental parameters

After the fabrication of a high-quality SPME fiber, the significant experimental parameters, such as extraction time, desorption solvent, desorption solvent volume, desorption time, pH of sample solution, stirring rate, and the salting-out effect have been optimized.

#### Extraction time

In the equilibrium-based sample preparation methods, the equilibrium time should be optimized to extract the maximum amount of the target analytes [46]. In the current work, different extraction time ranges from 5 to 30 min were studied. As it can be perceived from Additional file 3: Fig. S3A, the equilibrium has been reached at 15 min that indicating the fast adsorption kinetics of the NSAIDs on Zn-MOF-5@BHPAC sorbent.

#### Desorption solvent

Desorption of analytes from the fiber surface is a fundamental step in all SPME-LC systems. An appropriate desorption solvent should be strong enough to remove analytes from the fiber coating completely or as much as feasible [26]. Here the influence of the extraction time on the method efficiency was tested employing ethanol, methanol, acetonitrile, 1-octanol solvents. Based on the results (Additional file 3: Fig. S3B), methanol was chosen as the appropriate desorption solvent to obtain the highest signals intensity.

#### Desorption solvent volume

Large consumption of the desorption solvent presents a more efficient desorption process, minimizes the carry-over (memory) effect, and allows multiple injections of the same sample during the analysis. However, to reach the high sensitivities, the amount of the chosen desorption solvent should be as small as possible but adequate to immerse the fiber coating completely [47]. To determine the influences of this parameter, methanol volumes changed 1 to 2.5 mL. The results represent the maximum chromatographic signals obtained using 2 mL of methanol (Additional file 3: Fig. S3C).

#### Desorption time

Desorption time could influence the sufficiently release of the analytes from the fiber surface and avoid (or minimize) the carry-over affects [48]. The effect of desorption time on the extraction efficiency was estimated from 1 to 15 min, and the results are displayed in Additional file 3: Fig. S3D. It found that 5 min was sufficient to desorb all the selected drugs from the fiber surface.

#### Sample solution pH

The pH of sample solution can change the ionization form and the stability of analytes. Generally, the extraction output of acidic and basic compounds is strongly dependent on the pH of sample solutions. Some analytes van only be extracted quantitatively, if they present in the neutral state. In addition, the pH of the extraction mixture is notably critical for compounds having pH dependent dissociable groups [49]. Moreover, the pH can affect the substrate durability and coating materials stability [50]. Here, this factor was evaluated in the pH ranges of 1–11. Diluted HCl and NaOH have been employed for the pH adjustment. The results displayed that the best recoveries obtained at pH = 3 (Additional file 3: Fig. S3E).

#### Stirring rate

Usually, the agitation of the sample solution enhances the analyses’ mass transfer and extraction efficiency and consequently reduces the time required to reach equilibrium [51]. Here, the effect of the stirring rate was evaluated by analyzing the samples at various stirring rates from 0 to 1000 rpm. According to the results (Additional file 3: Fig. S3F), the stirring rate of the final experiments set at 800 rpm.

#### Salting-out effect

Salt is usually added to the aqueous sample in different sample preparation methods. The salt improves the extraction capability through the enhancing of ionic strength and decreasing the interaction of the analytes with water, and forces more analytes to go to the fiber surface [52]. For this determination, different quantities of NaCl were added to the sample solutions. The results demonstrated that the addition of 5% (w/v) NaCl to the samples increased the extraction efficiency (Additional file 3: Fig. S3G). Although after withdrawing the fiber from the solution, the coating material stripped and fell off. In other words, the salt destroys the fiber coating and makes it obsolete. Accordingly, all the analyses were performed without adding any salt.

### Carry-over effect

Typically, the carry-over effect is not a critical agent in the SPME procedure. However, at the very low concentrations of analytes, on-fiber carry-over may create a significant problem [53]. For the examination of the carry-over factor, a secondary desorption step (without any addition of analytes) was consecutively performed after the main desorption step. No obvious carry-over has been recognized in the results (Additional file 4: Fig. S4). It means that the analytes were desorbed completely in the first desorption step. Accordingly, no pre-conditioning was needed before the subsequent analysis, and the fibers were only washed with DDW to remove the possible contamination and solvent residue.

### Validation of the proposed method

The performance of the introduced method was evaluated under the optimized conditions and the results are expressed in Table 3. The method conferred good linearity over the range of 0.20–380 µg L^−1^ with a correlation coefficient (R^2^) more than 0.991. The linearity of calibration curves was studied utilizing Mandel’s fitting test. Mandel’s fitting test is determined with the difference of variance of the residual standard deviation of linear (Sy_1_) and the potential second-order (Sy_2_) calibration models. This is compared to the standard deviation of the potential second-order calibration model using the F-test (Eq. 2).

**Table 3:** Analytical figures of merit of the suggested method obtained using Zn-MOF-5@BHPAC nanocomposite-coated SPME fiber (n = 6)

Analytical figures	Analytes				
	Ketoprofen	Naproxen	Diclofenac	Ibuprofen	Mefenamic acid
LR^a^	0.20‒380	0.20‒300	0.5‒250	0.20‒380	0.40‒300
R^2 b^	0.9935	0.9928	0. 9936	0.9920	0.9913
LOD^c^ (µg L^−1^)	0.06	0.06	0.15	0.06	0.08
LOQ^d^ (µg L^−1^)	0.20	0.20	0.50	0.20	0.40
RSD^e^ of intra-day (%)	5.60	2.75	4.10	3.25	6.20
RSD of inter-day (%)	3.95	1.40	2.50	1.85	5.70
RSD after 45 days (%)	1.25	0.75	1.05	0.38	2.59
RSD of single fiber (%)	4.22	3.05	5.24	6.71	6.95
RSD of Repr.^f^ (%)	2.80	1.15	2.01	0.90	5.46
RSD of RF^g^ (%)	3.65	1.32	2.90	1.25	4.30
EF^h^	115	138	98	129	118

^a^Linear range

^b^Square of correlation coefficient

^c^Limit of detection

^d^Limit of quantification

^e^Relative standard deviation

^f^Reproducibility

^g^Response factor

^h^Enrichment factor

2 $${F}_{experimental}=\frac{{{S}^{2}}_{y1}\times \left(n-2\right)-{{S}^{2}}_{y2}\times (n-3)}{{{S}^{2}}_{y2}}$$

Here, the F_experimenal_ (in the confidence level of P = 95%) was calculated with the obtained experimental calibration results and the calibration functions. A comparison of the F_experimenal_ with the corresponding critical values (F-distribution tables) proved that using the second-order function did not create extraordinary better results. Consequently, the linear calibration function has been employed in this work. Accordingly, the calibration curve was obtained by plotting peak areas versus the spiked concentration of the desired drug in the real samples. Then the results were calculated by the linear regression model (Eq. 3), where y, a, x, and b are the instrument response, slope, nominal concentration of the drug, and intercept, respectively.

3 $$y=ax+b$$

To the study of method sensitivity, the limit of detection (LOD) and limit of quantification (LOQ) were calculated based on the six repeated analyses of the blank samples. The procedure offered LOD and LOQ in the range of 0.06–0.15 and 0.20–0.50 μg L^‒1^, respectively. The intra-day and inter-day relative standard deviations (RSDs) were also estimated (n = 6), lower than 6.20% and 5.70%, respectively. In addition, six new fibers were prepared on the same day and in the same conditions. The extraction and adsorption performance of these fibers was evaluated after 45 days. The results possess no reduction in the method efficiency. The RSDs value of these six fibers was in the range of 0.38‒2.59%. The RSDs of the response factor (RF, defined as the ratio between the concentration of an element and the related detector response) was also calculated (Eq. 4). RF is mainly employed to correct the difference between the detector response for impurities and analytes. Here, the RSDs of RF for analyzed drugs spanned between 1.25 and 4.30%.

4 $$RF= \frac{Peak\, area}{Concentration}$$

Moreover, the RSDs values of the fiber's reproducibility were in the range of 0.90–5.46 µg L^−1^. The reusability studies of the single fiber displayed no significant decrease in the recovery results, up to 80-repetitive adsorption–desorption cycles. After that, fiber’s efficiency has been diminished by about 5.92%. However, in the present study, each fiber was not utilized more than 10 times, to acquire good recoveries and repeatable outcomes. This means that the Zn-MOF-5@BHPAC nanocomposite coated fiber possesses a long loss-free service lifetime. Although in order to achieve good recoveries and repeatable results, none of the fibers was employed more than 10 times. The method enrichment factor (EF) [base on the ratio between the final analyte concentration in the extraction phase (C_f_) and the initial concentration of analyte (C_i_) (Eq. 5)] was in the range of 98–138.

5 $$EF= \frac{Cf}{Ci}$$

All of these results confirm the offered method’s robustness.

### Real sample analysis

To investigate the proposed fiber’s practicability and validity, the prepared blank and spiked (5.00 and 10.00 µg L^−1^) real samples (water and soil) were analyzed by the developed procedure (n = 3). The calculated recoveries and RSD values are summarized in Table 4. Based on the results, the determination of the target drugs from the blank/spiked samples submitted yields of more than 85%, indicating a method with good accuracy. The obtained recoveries (85.44‒103.73%) confirm that the method was not affected by the samples matrix. In total, the results support that the submitted method can be successfully utilize for the determination of pharmaceutical compounds in different samples with adequate accuracy and precision.

**Table 4:** The relative recoveries of NSAIDs determination by the prepared Zn-MOF-5@BHPAC nanocomposite-coated SPME fiber (n = 3)

Analytes	Added (µg L^−1^)	Powerhouse water		River water		Road soil		Beach soil	
		Found (µg L^−1^)	RR^a^ ± RSD (%)	Found (µg L^−1^)	RR ± RSD (%)	Found (µg L^−1^)	RR ± RSD (%)	Found (µg L^−1^)	RR ± RSD (%)
Ketoprofen	0.00	–	–	2.60	‒	5.19	‒	3.48	‒
	5.00	5.01	100.20 ± 4	7.37	91.15 ± 7	9.63	89.21 ± 3	8.00	86.20 ± 3
	10.00	9.84	98.40 ± 3	12.45	94.23 ± 3	15.00	96.33 ± 2	13.61	103.73 ± 4
Naproxen	0.00	‒	‒	8.90	‒	13.42	‒	7.07	‒
	5.00	4.86	97.20 ± 3	13.50	95.50 ± 2	16.62	86.58 ± 7	12.60	98.70 ± 5
	10.00	9.83	98.30 ± 6	18.93	100.33 ± 5	22.60	93.88 ± 3	17.00	90.90 ± 6
Diclofenac	0.00	‒	‒	6.55	‒	4.19	‒	‒	‒
	5.00	4.92	98.40 ± 5	11.02	91.90 ± 6	8.58	85.44 ± 5	4.80	96.00 ± 4
	10.00	9.50	95.00 ± 3	15.86	89.46 ± 3	14.01	97.85 ± 5	9.92	99.20 ± 7
Ibuprofen	0.00	‒	‒	10.03	‒	17.39	‒	8.83	‒
	5.00	4.60	92.00 ± 7	13.8	87.83 ± 5	22.20	96.20 ± 2	12.92	89.69 ± 5
	10.00	10.20	102.00 ± 2	19.5	94.71 ± 4	28.00	89.21 ± 4	18.25	93.43 ± 5
Mefenac acid	0.00	‒	‒	3.33	‒	5.70	96.33 ± 5	5.95	‒
	5.00	4.73	94.60 ± 3	8.00	90.09 ± 2	9.96	85.20 ± 3	10.22	87.73 ± 2
	10.00	9.52	95.20 ± 3	13.37	101.20 ± 2	15.71	86.58 ± 7	15.81	97.64 ± 3

– ND

^a^Relative recovery

The representative chromatograms of blank/spiked road soil samples are manifested in Fig. 6. These chromatograms expose the good cleanup ability of the offered method to determine the concentration of analytes in different real samples. The obtained results for the spike soil samples are also in reasonable compliance with the corresponding values. In addition, the chromatographic runtime was less than 20 min that permits to analysis large number of samples in a short period.

### Robustness of the suggested technique

The thermal and chemical stability of the proposed Zn-MOF-5@BHPAC nanocomposite coated fiber has been estimated in different conditions. The thermal stability has been examined by storing a few coated fibers at different temperatures ranging from 0 to 200 °C for 2 h. The chemical stability, adhesion, and swelling behavior of the Zn-MOF-5@BHPAC coating have been examined by dipping a few coated fibers in different media including deionized water, acetonitrile, ethanol, methanol, NaCl solution, KCl solution, acetone, n-hexane, and THF for 6 h. Except for NaCl and KCl, no swelling and stripping of the coating materials have been detected in other cases. In other words, the addition of salt to the sample solution damages the fiber sorbent and sharply reduces its lifetime and efficiency. These results have been proved that the synthesized nanocomposite holds its extraction capability in many harsh conditions. In addition, no stripping of the coating materials from the fiber surface was observed over time (even after several uses), during the sample stirring, and during the possible hits or contact with different objects. Functional groups of the prepared bread hydrochar will anchor the zinc oxide clusters and form a Zn-MOF-5@BHPAC hybrid nanocomposite. Then the prepared nanocomposite is chemically connected to the silanol-functionalized stainless steel to create a strong adhesion between the sorbent and the metal substrate.

### Comparison with other procedures

Employing green and affordable biomass (bread) to prepare a novel SPME fiber coating is the main innovation of this research work. The prepared BHPAC has been created a new high-capacity sorbent providing better properties than the similarly porous sorbents. In addition, its composite with Zn-MOF-5 was demonstrated good potential applications in the adsorption, extraction, and preconcentration process. Besides, utilizing solid wastes consist bread-wastes can efficiently save landfill space and enhance the biomass sources value. The offered coating material has not been investigated before and can be considered a novelty in the SPME fibers fabrication. Some statistical data of the proposed method were compared with similar and non-similar methods from some works of literature (Table 5). As can be observed, the developed method gives comparable or better proficiency with the other methods. In addition, a comparison of the proposed procedure significances with the other similar MOF/AC studies is displayed in Table 6. The advantages of the presented technique are clearly obvious from these results. Therefore, utilizing the Zn-MOF-5/bread biomass as a new sorbent promotes the creation of a highly efficient, low-priced, and green SPME fiber coating material that can be successfully employed for the routine analysis of the target drugs.

**Table 5:** Comparison of the performance of prepared Zn-MOF-5@BHPAC nanocomposite-coated SPME fiber with other NSAIDs determination methods

Analytical technique	Samples	LR (µg L^−1^)	LOD (µg L^−1^)	RSD%	Extraction time (min)	References
Electromembrane extraction-HPLC-UV	Urine	0.68–8.40	0.20–0.27	2.71–10.20	10	[54]
DLLME-SFO-HPLC-UV	Wastewater	1.00‒100	0.07‒0.19	3.05‒6.25	10	[55]
C_18_-SBSE-HPLC-UV	Water	20.00‒2000	6.90‒7.69	4.10‒9.20	60	[56]
Zr‑MOF@GO-SPME-GC-FID	Water	0.01–500	0.001–0.030	3.71‒3.77	40	[57]
Zn-MOF-5@BHPAC-SPME-HPLC-UV	Water Soil	0.20‒380	0.06‒0.15	1.40‒5.70	15	This work

**Table 6:** Comparison of the proposed procedure significances with the other similar MOF/AC studies

Composite	AC sources	Application	BET surface area of AC (m^2^/g)	Average nanocomposite synthesis time (h)	Refs
Fe-BDC@Biowaste AC	Cinnamon sticks	Adsorption	780	70	[23]
Zn-MOF@Biowaste AC	Gingko barks	Photocatalytic degradability	1753	12	[58]
STAM-17-OEt@BPL AC	Un-impregnated coal	Removal of ammonia	1209	80	[25]
NiO-MOF@Biowaste AC	Fallen waste leaves	Electrocatalysis	–	80	[59]
AC@NH2-MIL-101(Cr)	Commercial AC	Adsorption	2034	45	[60]
Zn-MOF@Biowaste AC	Bread	Extraction and adsorption	2158	45	This work

– Not mentioned

## Conclusion

To sum up, a simple and reliable DI-SPME-HPLC method utilizing a Zn-MOF-5@BHPAC nanocomposite-coated SPME fiber is introduced in the present study. The novel high-performance porous hydrochar was synthesized from the wasted bread within a suitable hydrothermal carbonization process followed by a chemical activation step. Then this carbonaceous material was hybrid with a Zn-based MOF compound to create a new high-capacity nanocomposite. The synthesized nanocomposite was attached to the surface of a stainless steel wire with epoxy glue and used as an SPME fiber. The method capability was examined by the simultaneous determination of ketoprofen, naproxen, diclofenac, ibuprofen, and mefenamic acid from different samples. Long service lifetime, economic efficiency, environmental frilliness, high adsorption capacity, good extraction capability, easy operation, fitting with the green analytical chemistry principles, and also acceptable precision, accuracy, and linearity are some of the main points of the proposed technique. In addition, the method possesses a relatively short runtime (20 min) that allows the routine quantifying of a large number of samples consisting of all the target analytes. There is hope that the recommended SPME-HPLC technique based on the proposed nanocomposite aid future efforts in routine analysis of the target drugs in the real world.

## Supplementary Information

**Additional file 1: Figure S1.** The effect of important parameters on the preparation of (A) BH, (B) BHPC, (C) BHPAC, and (D) Zn-MOF-5@BHPAC nanocomposite.

**Additional file 2: Figure S2.** The effect of important parameters on the Zn-MOF-5@BHPAC nanocomposite-coated SPME fiber fabrication: (A) kind of fiber coating, (B) etching reagent, (C) etching time, and (D) number of coating layer.

**Additional file 3: Figure S3.** The effect of different experimental conditions on the recommended method efficiency, achieved by the prepared Zn-MOF-5@BHPAC nanocomposite-coated SPME fiber: (A) extraction time (B) desorption solvent, (C) desorption solvent volume, (D) desorption time, (E) pH of sample solution, (F) stirring rate, and (G) salting out effect.

**Additional file 4: Figure S4.** Evaluation of the carry-over effect at the optimized conditions

## Data Availability

Adequate and clear descriptions of the applied materials and tools are provided in the materials and method section of manuscript. In addition, the obtained data is clearly justified by mentioning the figures and tables in the manuscript.
