# Supplementary figures and images for: Introduction of a Zn-based metal–organic framework @ biomass porous activated carbon as a high-sensitive coating for a stainless steel SPME fiber: application to the simultaneous analysis of nonsteroidal anti-inflammatory drugs
Source: BMC Chem. 2022 Apr 5;16(1):25. doi: 10.1186/s13065-022-00818-w (PMC8985354; doi:10.1186/s13065-022-00818-w)

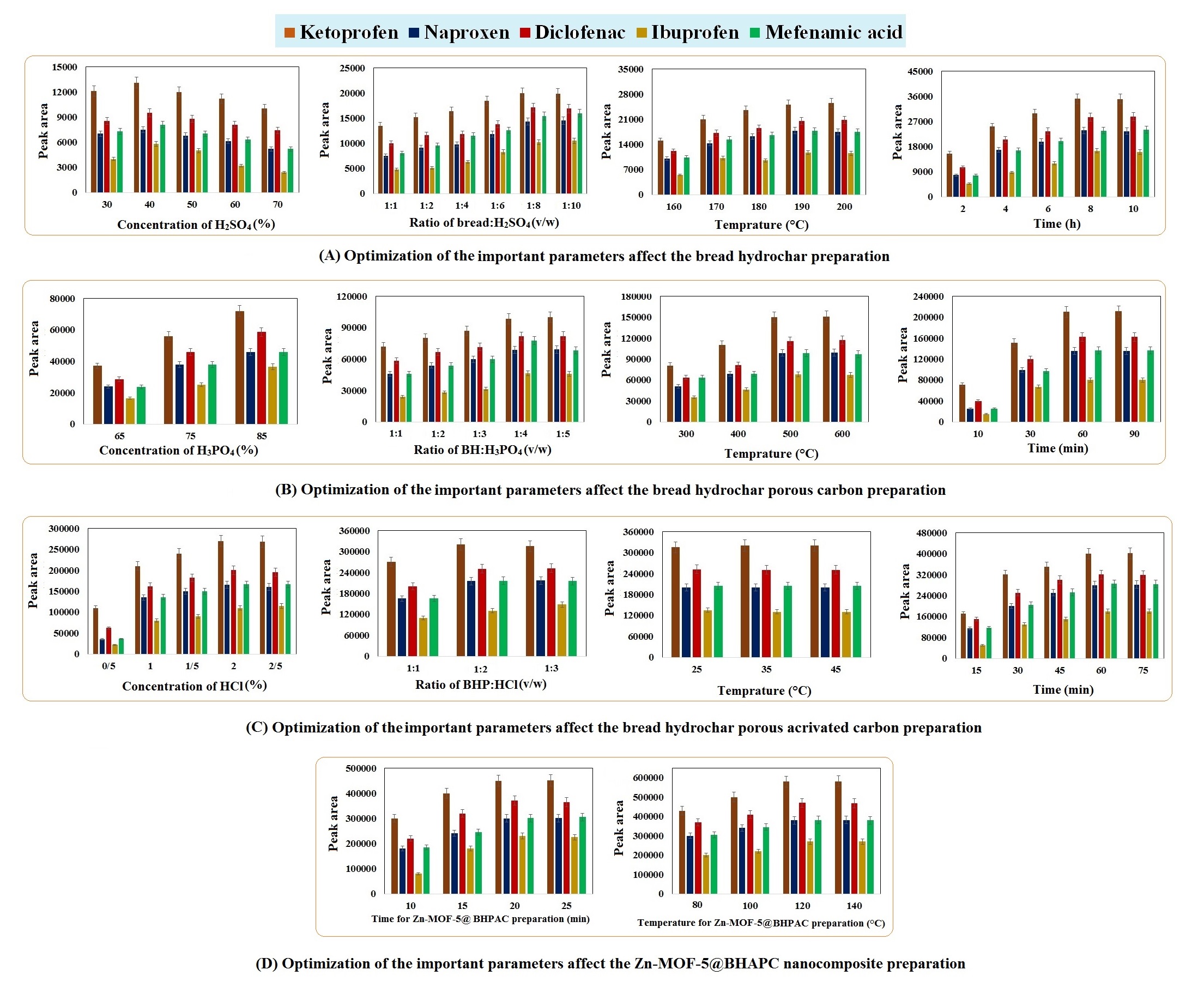

Supplement: Supplementary file 1 — Additional file 1: Figure S1. The effect of important parameters on the preparation of (A) BH, (B) BHPC, (C) BHPAC, and (D) Zn-MOF-5@BHPAC nanocomposite. [file 13065_2022_818_MOESM1_ESM.jpg]

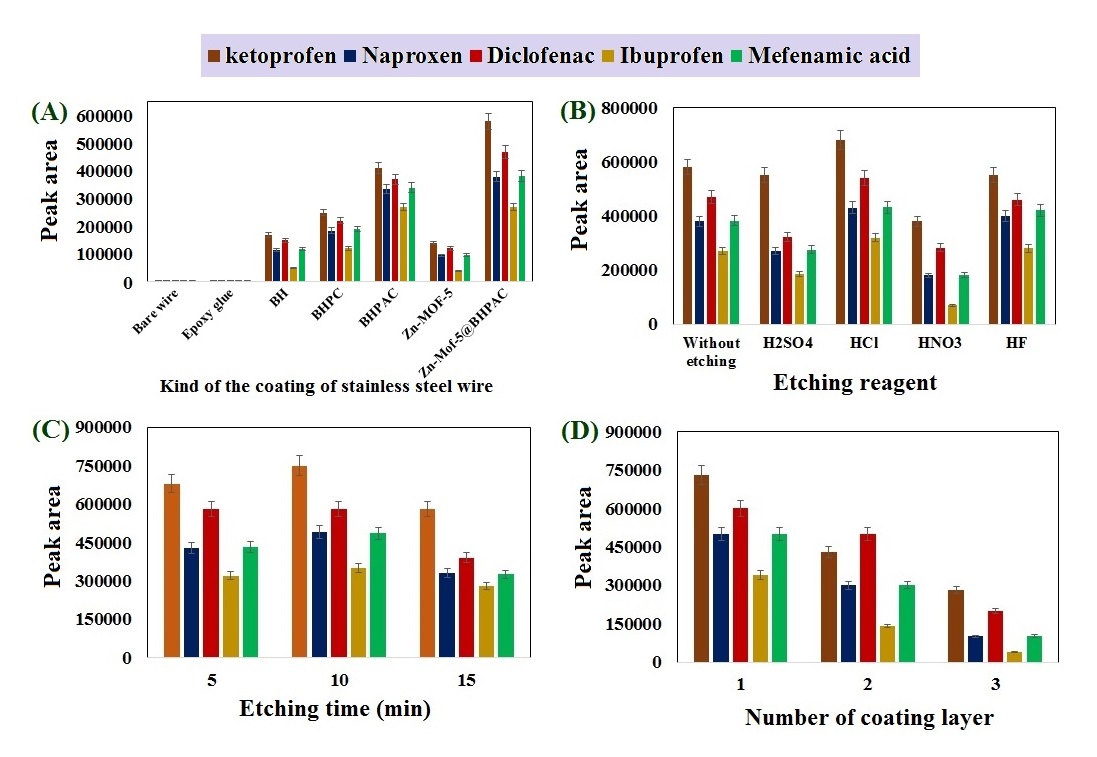

Supplement: Supplementary file 2 — Additional file 2: Figure S2. The effect of important parameters on the Zn-MOF-5@BHPAC nanocomposite-coated SPME fiber fabrication: (A) kind of fiber coating, (B) etching reagent, (C) etching time, and (D) number of coating layer. [file 13065_2022_818_MOESM2_ESM.jpg]

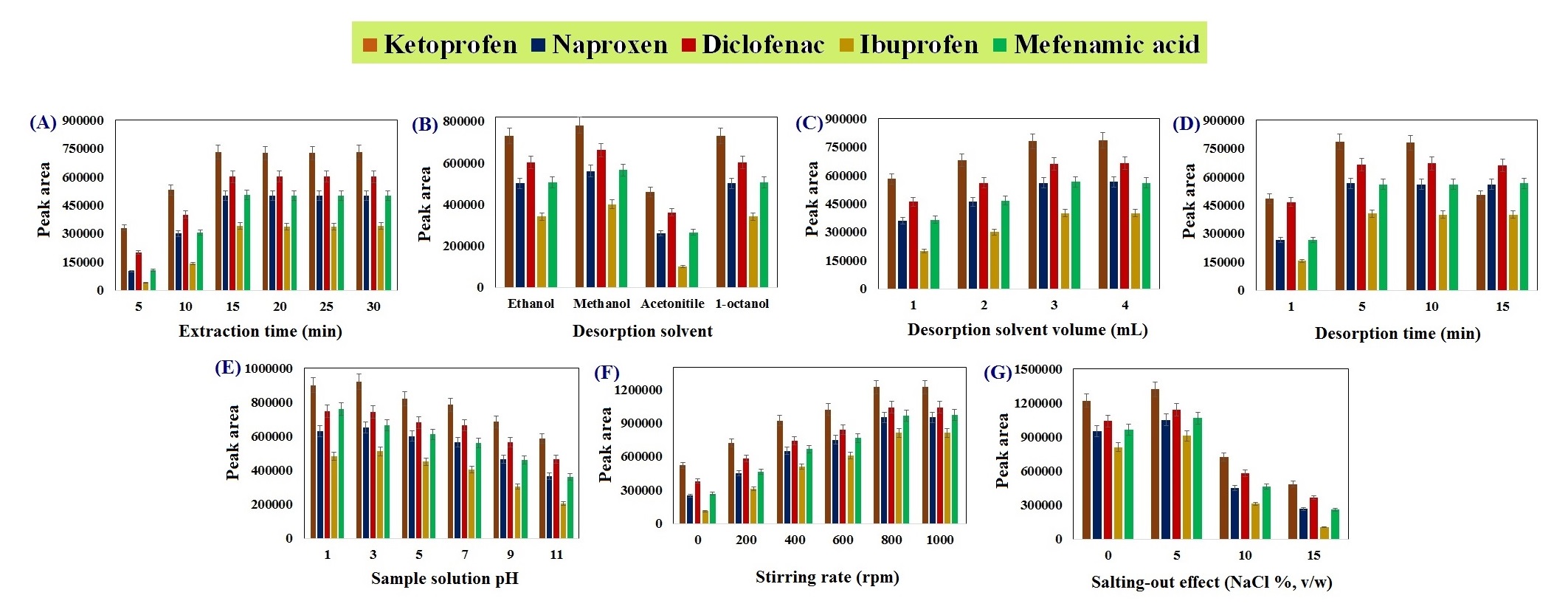

Supplement: Supplementary file 3 — Additional file 3: Figure S3. The effect of different experimental conditions on the recommended method efficiency, achieved by the prepared Zn-MOF-5@BHPAC nanocomposite-coated SPME fiber: (A) extraction time (B) desorption solvent, (C) desorption solvent volume, (D) desorption time, (E) pH of sample solution, (F) stirring rate, and (G) salting out effect. [file 13065_2022_818_MOESM3_ESM.jpg]

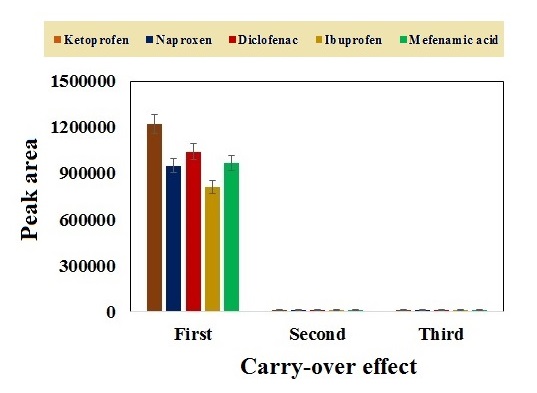

Supplement: Supplementary file 4 — Additional file 4: Figure S4. Evaluation of the carry-over effect at the optimized conditions [file 13065_2022_818_MOESM4_ESM.jpg]
